# Basic Characteristics and Spatial Patterns of Pseudo-Settlements—Taking Dalian as An Example

**DOI:** 10.3390/ijerph13010145

**Published:** 2016-01-20

**Authors:** Jiaji Gao, Yingjia Zhang, Xueming Li

**Affiliations:** 1 School of Urban and Environmental Sciences, Liaoning Normal University, Dalian 116029, China; gjj@dlou.edu.cn (J.G.); zyj575657@163.com (Y.Z.); 2 Applied Technology College of Dalian Ocean University, Dalian 116300, China; 3 The Research Center of Human Settlements, Liaoning Normal University, Dalian 116029, China

**Keywords:** pseudo-settlement, spatial pattern, characteristics, Dalian

## Abstract

A person’s living behavior patterns are closely related to three types of settlements: real-life settlements, imagined settlements, and pseudo-settlements. The term “pseudo-settlement” (PS) refers to the places that are selectively recorded and represented after the mass media chose and restructure the residence information. As the mass media rapidly develops and people’s way of obtaining information gradually change, PS has already become one of the main ways for people to recognize and understand real-life settlements, as well as describe their impressions of imagined settlements. PS also has a profound impact on tourism, employment, investment, migration, real estate development, *etc.* Thus, the study of PSs has important theoretical and practical significance. This paper proposes to put forward residential quarters where the mass media is displayed as the object of study and establishes the pseudo-settlement index system of Dalian in and elaborate analysis of the concept of PSs. From three aspects, including pseudo-buildings, pseudo-districts and pseudo-culture, this paper uses the ArcGIS 10.0 kernel density (spacial analyst) to analyze and interpret the basic characteristics and spatial patterns of 14 elements of the PS in Dalian. Through systemic clustering analysis, it identifies eight major types of PSs in Dalian. Then it systematically elaborates current situations and characteristics of the spatial pattern of PSs in Dalian, namely: regionally concentrated, widely scattered and blank spaces without pseudo-settlements. Finally, this paper discusses the mechanism of formation of PSs in Dalian.

## 1. Introduction

Space-time urban geography theory and empirical studies have confirmed that the range, scope, vigor and attention of people’s daily lives are limited. Thus, it is impossible for people to maintain full empirical contacts with their related external urban environment [1]. Living in a vast and complex modern urban society, people rely on traditional media outlets (television, radio, the Internet, billboards, *etc.*), social network new media, real-life social circles and other news sources increasingly to recognize and understand the world, which is just like the proposal made by the pseudo-environment master, Li Puman [2,3] “some people have told us what the outside world is like before we observe the world, and for most things, we first imagine these things and then experience them”. On these grounds, urban settlements are divided into three types; real-life settlements and imagined settlements, which people imagine in their minds, and pseudo-settlements, displayed by various media (as shown in Table 1).

**Table 1:** Explanations to concepts related.

Real-Life Settlements	Places People Work, Live, Rest and Socialize in Real-Life
Pseudo-Settlements	Places that are selectively recorded and re-presented after the mass media chose and re-structured the residence information.
Imagined Settlements	Places that people imagine after rational thinking and perceptual cognizance based on the information they got from real-life settlements and pseudo-settlements.
Spreaders	People who collect, make and spread information. It is also possible that spreaders use the mass media to direct the choice of audiences for certain purposes.
Pseudo-Display	All the information spreaders selectively choose, code and spread.
Audience	People who receive the information from mass media. They are one of the reasons spreaders spread information and the object pseudo-settlements affects.

Pseudo-settlements are relatively complicated information-spreading activities. They involve three elements: spreaders, pseudo-display and audiences (as shown in Table 1). Spreaders are people who collect, make and spread information. It is also possible that spreaders use the mass media to direct the choice of audiences for certain purposes; the audience are people who receive the information from mass media. They are one of the reasons spreaders spread information and the object pseudo-settlements affects; pseudo-display is all the information spreaders selectively choose, code and spread. As the mass media develops, people can receive massive information from PSs in forms of signs, audios, videos, photographs, words, *etc.* through the Internet, television, movies, magazines, books, *etc.* People’s cognition, behaviors and decisions are closely related to the PSs. The foundation of PSs are the combination of real-life settlements and imagined settlements, which are the subjective and objective settlements. Real-life settlements are the realistic foundation and the imagined settlements are the psychological foundation. In order to form effective pseudo-displays, spreaders need to have a fully understanding of the realistic foundation and evaluate audiences’ psychological foundation. Based on audiences’ political, economic intentions and value, spreaders synthesize audiences’ real-life settlements and imagined settlements and selectively choose and edit information. Eventually, the spreaders spread symbolic words and signs to audiences through mass media. The purpose of pseudo-displays is to form dynamic reflections to real-life settlements to influence and optimize audiences’ cognition, attitudes, emotions and expectations towards real-life settlements, and on these grounds, form new kinds of imagined settlements. PS is a selective record and representation of real-life settlements, it is also a crucial information source for imagined settlements. PS is not only a kind of information exists independently, but also a bridge that links real-life settlements and imagined settlements. The ways that human perceive the world have gradually shifted from perception of a real environment to the perception of a pseudo-environment which was created by the media. A pseudo-settlement is a human settlement environment presented by the media through processing some symbolic events or selected information. In other words, people perceive the world according to the information presented by the media and jumping off the whole appearance of the real human settlement. The information dissemination and audience’s personal interpretation can induce the deviation between pseudo-settlement and real-life settlement.

Geographers have performed research on real-life settlements [4], while PS and spatial structure have always been the weak points of the study. However, the image map [5] made by Lynch represents a significant breakthrough [6] in the study of the urban spatial imagination. Geographers can now depict people’s direct feelings through the imagination map by using visualization methods and apply them to fields like urban tourism planning [7], urban designing [8], residential quarter planning [9,10], *etc.* PS is still a field that the academia needs to breakthrough. The concept of PS originated from Lippman [11] and he pointed out that the media provides information that is a major component of our “pictures”, also termed this a pseudo-environment. Cameron [12,13] came up with the concept of pseudo-community and developed it, promoted pseudo-community to acquire deeper roots and greater usefulness. Boorstin [14] defined pseudo-events as news planned for the purpose of being reported. For the conveniency of the media doing the reports, Beniger [15] demonstrated the growth of pseudo-communities under the circumstance of personalization of mass media. The news terminology “pseudo-settlement” gained attention from scholars in different fields. For the past few years, the amount of related research has kept growing, Clarke [16] observed that the key to the success of pseudo-events is that journalists are willing to report them. Journalists may be more dramatic, they may be less complex and therefore easier for audiences to understand. Politically, Paterson [17] sought to provide insight into Kosovo’s declaration of the independence as a pseudo-event. Among the celebrities, Hillman [18] made a case for the importance of celebrity formations and performances. Also, tourism related, Nedelea [19] pointed out that scholars must draw the attention towards the proliferation of “pseudo-tourists”.

Pseudo-settlements are still growing in the disciplines of news media, psychology, architecture [20,21,22,23], *etc.*, but few people are involved in the field of geography. This paper takes Dalian as an example, using the Internet, magazines, advertisements, maps, *etc.* to collect information about residential quarters’ names to demonstrate PSs in Dalian. The study of the spatial characteristics of PS expands the study scope of human settlement and deepens the relationship among the media, people and city. It seeks to analyze urban settlements from a more humanized angle of view, and plays an important theoretical and practical role in the study of urban pseudo-settlements.

## 2. Study Object and Index System

### 2.1. Study Object

There are different forms of PSs, and the selection of names of residential quarters as the study object is based on the following four reasons: (1) Names of residential quarters are the most concentrated pseudo-exhibition and expression of human settlement of real districts. Great importance is attached to naming in Chinese culture. Chinese uses an ideographic writing scheme, thus the names of people, stores and places are not only the pure symbols of names, but also give a variety of cultural connotations and value concepts to them [24,25]. During planned economy period, houses were allocated by the government, and the built communities were mostly given names such as “family dormitory buildings”, “residential quarters”, “welfare districts” and “new villages”, which mainly showed the work unit’s residential quarters and street jurisdiction scope. With the development of the real estate market, the competition in this market has gradually become fiercer. During the time of the development of various residential quarters, in order to build the developers’ brands and attract target customers, real estate developers constantly contemplate and design the names of residential quarters to have appropriate implications and deep coverage to match with the geographical location, surrounding environment, regional features, house quality, residential culture, historical context, brand image, *etc.* of the residential quarters; (2) The names of residential quarters are the functional and indicative names obtained after repetitive conception, and thinking. The names of residential quarters belong to the category of geographic names, and they are a kind of language code given to the residential quarters which are the geographic entity in the specific orientation and scope in cities. They are public names officially approved and filed by a specific government department. The transmission contents in the form of images and videos, *etc.* can show a visual effect of PS, but usually the audience cannot confirm the spatial location of the PSs if there is no text description, and these PSs can also be easily mix up with similar PSs; (3) The name of a residential quarter is one of the main sources from which people can obtain the first impression of the human settlement of this residential quarter. And the name will be transmitted broadly and enduringly. Upon filing in the specific government department, the name of the residential quarter will be used in real estate planning, marketing, purchasing, checking in and a series of subsequent activities. As the main content in a variety of real estate advertising media, the name of residential quarter occupies a large and obvious area on advertisements, and the promotion is long-lasting and concentrated. The name of a residential quarter may be permanently recorded in official maps, electronic maps, books, bus stop names, *etc.*; (4) In comparison to signs, videos, photographs and words, the names of residential areas are easier to collect, arrange, count, calculate and analyze. Therefore, the name of a residential quarter is a study object with the highest occurrence frequency in PSs. It is the most direct, most obvious and easier to spread, has the highest concentration of real-life settlements, and related data can be easily gathered for the purpose of spatial analysis.

### 2.2. Study Area and Data

**Figure 1:**
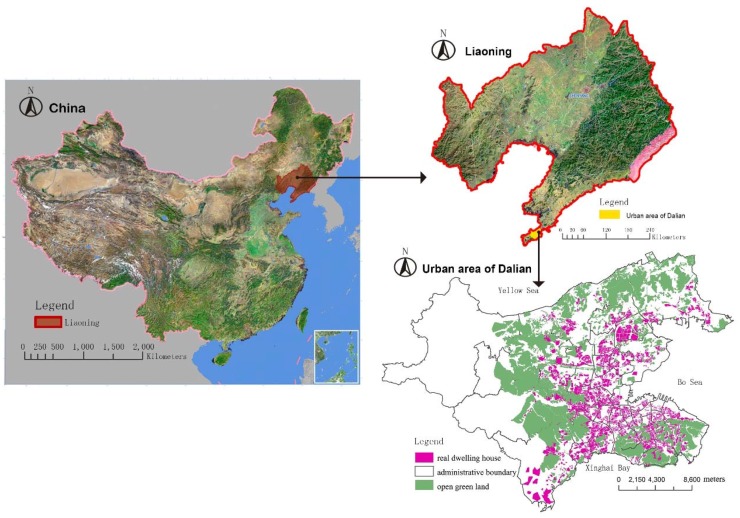
Map of the study area.

Dalian is an important economic, trade, port, tourist and coastal city. It is located on the southern tip of the Liaodong Peninsula in northeast China, with the Yellow Sea on the east, Bohai Sea on the west, the Shandong peninsula across the sea on the south, and the vast Northeast Plain on the north (Figure 1). The spatial data of residential quarters used in this paper is from the Land Resources Development Research Center of Dalian, and the names of the residential quarters are from Baidu Maps, Soso Maps, SouFun, Dalian Real Estate, Peninsula Morning News, and bus stop names (Table 2). A total of 806 names of residential quarters were collected from the main urban districts of Dalian, including Zhongshan District, Xigang District, Shahekou District, and Ganjingzi District.

**Table 2:** Data source illustration.

Data Classification	Data Source	Website
Residential Quarter Spatial Data	Land Resources Development Research Center of Dalian *	http://www.gtfwj.dl.gov.cn/
Residential Quarter Names	Baidu Maps	http://map.baidu.com/
	Soso Maps	http://map.qq.com/
	*Dalian Real Estate*	http://www.lowlo.cn/
	SouFun	http://dl.fang.com/
	Bus Stop Names	http://www.dalianbus.com/sngj/index_gjxl.asp
	*Peninsula Morning News*	http://epaper.hilizi.com/

* The spatial data is collected by Land Resources Development Research Center of Dalian, which is a public institute, responsible for information collection, scientific research and technical advice on land resources and real estate. The data is non-public and only used for nonprofit research.

### 2.3. Methods of Extracting and Distinguishing Characteristics of the Pseudo-Settlements in Dalian

First, the value of each residential settlement needs to be assigned to an index. The index value of the name of the residential quarter shall be 1, while the remaining index value is 0. Take Hongji Scholarly Park as an example; the assigned value to the index of the developer characteristic is 1 (Hongji), the allocated index value of campus culture (scholarly) is 1, the allocated value to the courtyard building cluster in courtyard is 1, and the remaining index value is 0. Furthermore, influential indices are included from the PS index in Dalian (its proportion is greater than 8%, namely there are more than 64 residential quarters complying with the index characteristics).

### 2.4. Visualization Methods for Dalian Urban Pseudo-Settlement

We use the ArcGIS10.0 (Environmental Systems Research Institute Inc.: RedLands, CA, USA) platform data management tool to turn the points of the faceted data elements of the Dalian residential quarters and we also adopt the GIS spatial analysis tool, kernel density analysis to demonstrate Dalian PS’s environmental characteristics and spacial patterns. Kernel density analysis is a process which uses discrete sampling points to conduct surface interpolation. The smooth lines in our charts identify and express the gathering and dispersion of samples in the study area. Kernel density searches in a circular area, regarding each calculation grid point as the center. During the process of calculation, the search range that closes in on the target point will have a larger proportion so that the density value of each grid point can be calculated, and a continuous density surface will be generated.

## 3. Results

### 3.1. Architectural Features of Pseudo-Settlements

Pseudo-architectural features include the appearance, style, and spatial structure of settlements, as well as the simulation and exhibition of architectural art. These pseudo-features are among the most important cultural features of the PS. The structures mainly consist of private and detached PSs, high-rise PSs, Large Building Cluster PSs and courtyard building cluster PSs (Table 3).

**Table 3:** Characteristics of pseudo-settlements in Dalian.

Pseudo Type	Characteristics of Different Categories	Sample Quantity/Ratio	Example
Architectural Characteristics	Private and Detached PSs	77/9.553%	Tiger Beach Villa, Golden Bauhinla Mansion, Fuji Manor, Zhongnanhai Palace
	High-rise PSs	97 /12.034%	Wanda Mansion, Plat Villa, Dalian Greenland Centter, New Tiandi Mall
	Large Building Cluster PSs	89 /11.042%	Peace Plaza, Top of the City, Wanghai Community, Fujia Mingdu
	Courtyard Building Cluster PSs	387/48.015%	China Yard, Buttonwood Garden,
Region Characteristics	Water-related PSs	169/20.968%	Sea of Love Garden, Seaview Imperial Garden, Haitian Yingzhou, Jinguang Coast
	Mountain-related PSs	95/11.787%	Beauty Peak East Park, Celestial Heights, Mountain Living Note, Sight Ocean
	Flower-related PSs	167/20.720%	Narcissus House, Sakurrrar House, Herbs Garden, Lotus Bay, Guanshan Butterfly Flower
	Street-related PSs	85/10.545%	Beijing Park, Liande District, Minxing Garden, Xinhua AZOasis
Cultural Characteristics	Urban Culture-related PSs	78/9.677%	City Ocean View, City Melody, Eton Place Dalian, Orstar City
	Western Culture-related PSs	66/8.169%	Fragrance of Vienna, Houses in California, International New City, European Town
	Science-related PSs	65/8.065%	Googleli, Silicon Valley Holiday, International Living Space, Eastern San Jose
	Family-related Cultural PSs	84/ 10.422%	Shanshuijiayuan (Landscape Homeland), Happy Family, Golden Sunshine Home, Hibiscus family
	Fortune-related Cultural PSs	123/15.261%	Gold Drill Treasure, Dalian Pearl, Blessed Mountain Household, Fuhua Mountain Villa, Wealthy Court
	Developer Brand Name-related PSs	160/19.851%	Fairview Garden, Wanda Mansion, Wanke City Garden, Classic Palais

Private and Detached PSs are mainly distributed in the Xinghai (Sea of Stars) Bay and Nanshan areas, due to their better landscape and higher real estate value. They also benefit from the outward expansion of Dalian real estate developers and the public’s yearning for private detached buildings and this shows a trend of focal layout and comprehensive popularization.

**Figure 2:**
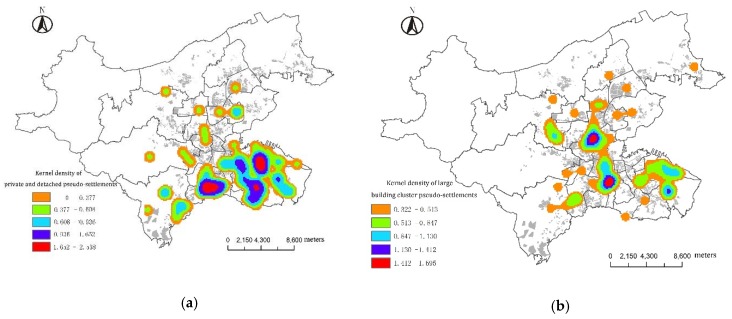

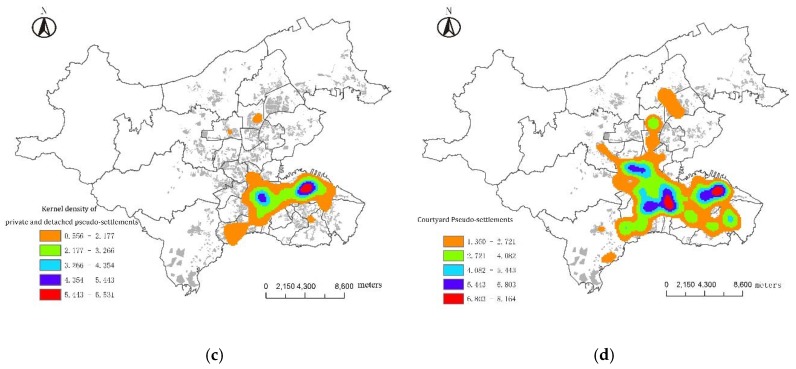
Spatial features of pseudo-architecture of human settlements in Dalian. (**a**) Private and detached pseudo-buildings; (**b**) High-rise pseudo-buildings; (**c**) Pseudo-building clusters in vast residential quarters; (**d**) Country pseudo-building clusters.

Dalian High-rise PSs are highly concentrated around the city’s CBD and sub-CBD areas. Dalian Large Building Cluster PSs are mainly distributed in the northern Ganjingzi District, western Ganjingzi District and eastern Zhongshan District. Courtyard building cluster PSs have the most extensive distribution and largest number of PSs (Figure 2).

### 3.2. Regional Features of Pseudo-Settlements

**Figure 3:**
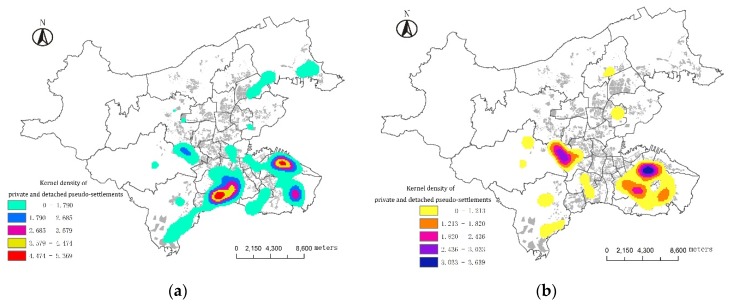

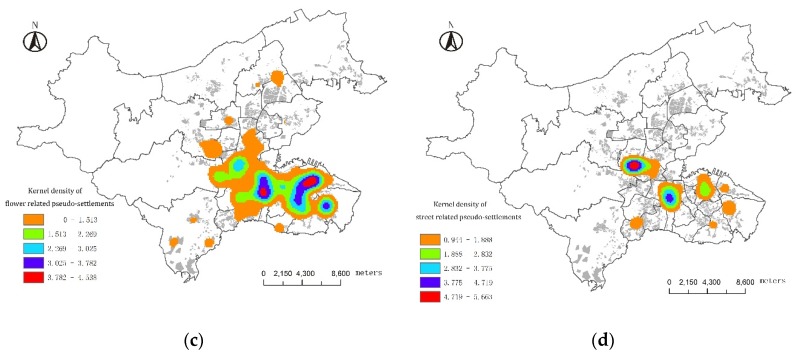
Spatial features of the pseudo-environment of human settlements in Dalian. (**a**) Water-related pseudo-settlements; (**b**) Mountain-related pseudo-settlements; (**c**) Flower-related pseudo-settlements; (**d**) Street-related pseudo-settlements.

Human settlements are the results between the interaction of human activities and natural environment. Dalian features a coastal hill landform where the idyllic PSs can attract the favor and yearning of the audience (Table 3). Closeness to the sea is the main feature of water-related PSs, but the settlements are not uniformly distributed along the coastal line. They are mainly distributed in Xinghai Bay, East Harbor and Tiger Beach which are the tourist areas and are also distributed in the northern part of Dalian Port. In addition, the West Mountain Reservoir in the west is also a water related sub-core pseudo-settlement area. Mountain-related PSs are mainly distributed in the West Mountain and South Mountain areas, which have significant landscape advantages, while other hilly and mountainous areas are sparse. Flower-related PSs usually use the names of plants as special names for residential quarters. This can not only bring people a pleasant and natural artistic conception, but can also bring people a warm and happy family atmosphere. They are mainly distributed in the Zhongshan District, Xigang District and other old districts. They mirror the PS characteristics in the 1980s–1990s. Human settlements are very sensitive to street sections, as some streets have fixed or biased images in the hearts of the audience. -elated PSs usually use the names of streets as the special name of residential quarters, such as Fengshang, Zhongnan (Road), Beijing (Street) Park, Jinxiu (Street) Garden, *etc.* (Figure 3).

### 3.3. Culture Features of Pseudo-Settlements

The diversity of residents’ culture, education, economic background and demands causes the diversified requirements of the living culture. The PS culture of residential quarters in Dalian mainly include Western culture, science and education culture, urban culture, developer brand culture, “Happy, Auspicious, Safe and Healthy” family culture, and “Water, House, “Zun and Fortune” culture (Table 3).

The urban cultural PSs in Dalian are mainly concentrated around the CBD and sub-CBD areas. Western culture PS are usually named using foreign place or foreign city names. Sometimes, they are named using words like “international” and “world” or Chinese names with English letters in between. The core areas are distributed in Dalian High-tech Zone and Dalian Zhongshan Square, but there is a tendency of expansion towards the northern Ganjingzi District.

**Figure 4:**
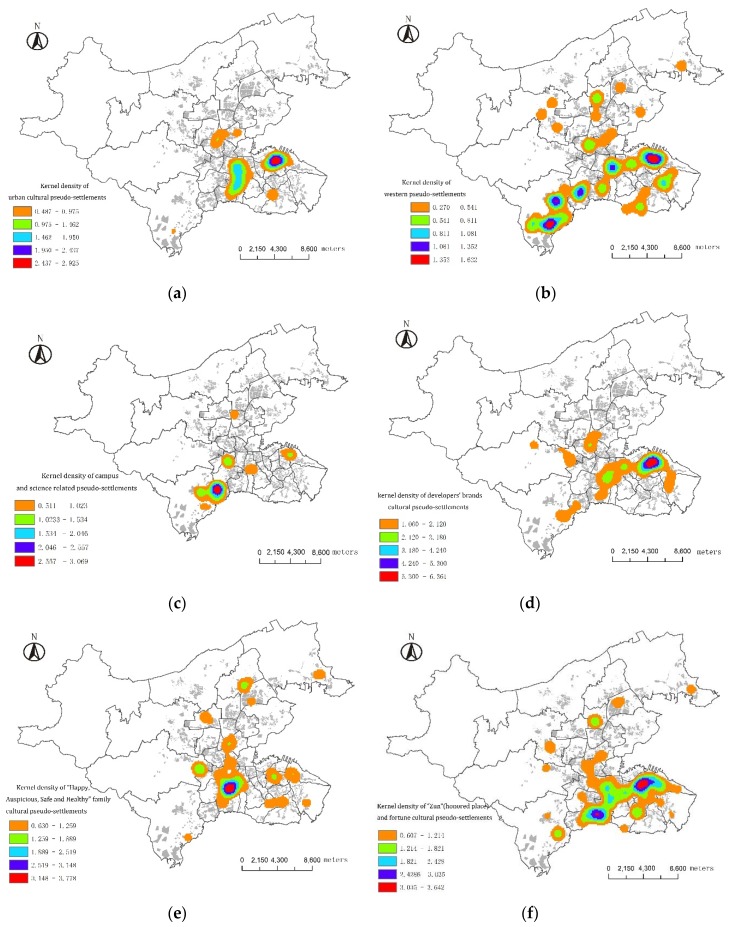
Spatial features of the pseudo-culture of human settlements in Dalian. (**a**) Urban culture-related pseudo-settlement; (**b**) Western culture-related pseudo-settlement; (**c**) Campus technology culture pseudo-settlements; (**d**) Developer brand name-related pseudo- settlements; (**e**) Family-related pseudo-settlements; (**f**) Water, house, “Zun” and fortune-related culture pseudo-settlements.

Campus- and science-related cultural PSs are usually named by using world-famous universities’ names or famous science and technology cities’ names, They are named in traditional cultural style, such as University Court, Literary Family, Mr. Wisdom, Wen Cui Xuan, *etc.*, while some are named in a straightforward style, such as Science and Technology Square, Academism, Yida Academy, Scholar Park, Scientists Apartments, *etc.* These PSs named in straightforward style are mainly distributed in Dalian High-tech Zone. In recent years, with increasing awareness of brands and the successful establishment of developer brand culture, more and more residential quarters are named with developer’s brands. They are mainly distributed in the central area of the city. In addition, Dalian High-tech Zone, Xishan District, *etc.* are also the preferences of developers’ brand culture. Chinese value family and “Happy, Auspicious, Safe and Healthy” family culture PSs contain the idea of the blessed family. The core area for this type is distributed in Shahekou District, and the distribution in other districts is sparse. Along with the rapid development of the Chinese economy, there has been an increase in the number of wealthy people. The significance of life success can be easily spread to the audience through the expression of “Water, House, Zun, and Fortune” cultural PSs. These PSs meet wealthy people’s imagination of a prosperous life. They are mainly found in Xinghai Bay and Zhongshan Square (Figure 4).

### 3.4. Spatial Patterns of Pseudo-Settlements in Dalian City

#### 3.4.1. Details of the Classification Method of the Spatial Patterns of Pseudo-Settlements in Dalian City

We used SPSS to select 14 influential indices as variables, and performed the system cluster analysis [26,27] of 806 samples in accordance with the squared Euclidean distance. After obtaining the results and tree diagram, we compiled statistics of 39 subdistricts in the main urban districts of Dalian. In accordance with the main types of subdistricts, we then divided the PSs in Dalian into eight types of PS districts.

#### 3.4.2. Spatial Patterns of Dalian Pseudo-Settlements

**Figure 5:**
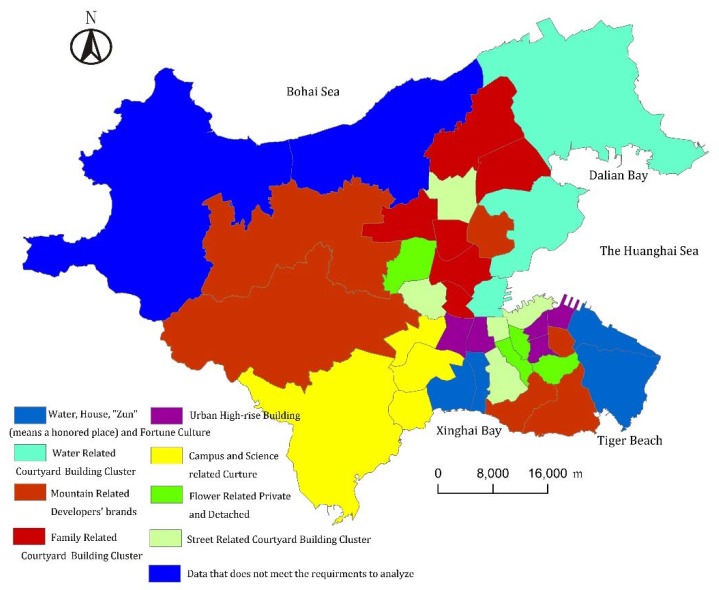
The spatial features of pseudo-settlements in Dalian.

There are eight types of PSs in Dalian, and their spatial patterns are as follows (Figure 5): (1) Water, house, “Zun” and fortune cultural PSs are located in Xinghai Bay Subdistrict in Shahekou District, as well as Tiger Beach and Navy Square Subdistrict in Zhongshan District. These PSs are highly dependent on the coastal landscape advantages of real-life settlements. These PSs meet the psychological needs of high-end audience by virtue of traffic and commercial advantages; (2) Courtyard building cluster PSs with words related to water and houses in its residential quarters’ names are located in Dalian Bay Subdistrict, Ganjingzi Subdistrict and Xianglujiao Subdistrict in Ganjingzi District along the northeast coast of Dalian. These places are seascape residential areas located in relatively distant locations, with needs for further improved city construction. They meet the desire of the middle class’ seascape preference; (3) Developer branded PSs with words related to mountains, houses and developers’ brand names in its residential quarters’ names rely on Dalian’s hills. They are emerging forms of PSs in districts of Xishan, Nanshan, Dalian Forest Park, Lotus Mountain, Jiaojinshan, *etc.* In the case of which the audience’s recognition degree of developers’ brands, the audience’s acceptance of mountain landscape and the shortage of available urban residential quarters are gradually increasing, these PSs are the mainstream ones recognized by the audience. The primary audience is young people, new migrants and young professionals. (4) Courtyard building cluster cultural PSs with words related to family in its residential quarters’ names are located in Chunliu Subdistrict, Nanguanling Subdistrict, Paoya Subdistrict, Quanshui Subdistrict, Xinghua Subdistrict and Zhoushuizi Subdistrict in the northern part of Ganjingzi District of Dalian. They are Dalian’s traditional industrial bases. They meet the desire of the working class’ imagination of a blessed family; (5) High-rise PSs with words related to the urban environment in its residential quarters’ names are concentrated around Qingniwaqiao Subdistrict, Renmin Road Subdistrict, Kunming Subdistrict around Qingniwaqiao Commercial Center in the CBD of Zhongshan District. These PSs also concentrated around Xinggong Subdistrict and Zhongshan Park Subdistrict in the sub-CBD of the Xi’an Road business circle in Shahekou District. PSs in these areas can meet the housing needs of modern white-collar and business people; (6) Cultural PSs with words related to campus and science in its residential quarters’ names are mainly located in the southwestern area of Dalian. High-tech Industry Park. International multi-cultural communication centers and universities are in that area. It covers a large area from the High-tech Zone of Dalian to the Heishijiao Subdistrict and Nansha Subdistrict in the Shahekou District, which can meet the housing needs of international personnel, scientific and technical workers, college teachers and some business people; (7) Private and detached PSs with words related to flowers in its residential quarters’ names are distributed around Rixin Subdistrict and People’s Square Subdistrict in Xigang District, as well as Kuiying Subdistrict in Zhongshan District. These PSs mainly rely on the landscapes, horticultural installations and its political center location of Renmin Square, Labor Park, Dalian Plant Park and Heroes Memorial Park areas. These locations have simple traffic and prosperous business, which can make residents’ life more convenient and meet the needs of high-salary populations. In addition, the Airport Subdistrict in Ganjingzi District relies on Xinle Garden and airport greening, *etc.*; (8) Courtyard building cluster PSs with words related to nearby streets’ names in its residential quarters’ names are mainly distributed around Northern Xigang Station Subdistrict, Baiyun Subdistrict and Beijing Subdistrict. Most of these subdistricts are modern residential areas with brilliant facilities built after the reconstruction of the old Dalian. These are the areas where local residents have a sense of belongingness and sense of identity. In addition, Zhonghua Road Subdistrict (Zhonghua Road, Zhonghua West Road, Huabei Road and Huadong Road) in Ganshizi District have places with names like Huagui Garden, Huacai Garden, Huachun Garden and Huali Garden (the word “Hua” means “luxurious” in Chinese). Also, Lijia Subdistrict (Jinxiu Subdistrict) in Shahekou District have places with names like Jinquan Garden, Jinxiu Garden, Jinxia Garden, Jinjiang Garden (the word “Jin” means “brocade” in Chinese).

#### 3.4.3. Spatial Pattern Characteristics of Pseudo-Settlements in Dalian

*Places without PSs*. The real-life settlements on Gezhenbao Subdistrict and Yingchengzi Subdistrict in the northern part of Ganjingzi District are mostly old dwellings. They have incomplete community functions and relatively poor public infrastructures. Moreover, they are also located far from the downtown area [28]. Therefore, they are areas with poor habitability [29]. The public, real estate developers and spreaders have very low interest in these areas. Thus, few names of residential quarters are spread through billboards, magazines, newspapers, internet, maps *etc.*, which cannot meet data analysis requirements. These areas in Dalian are areas without PSs.

*Widely scattered*. Different from the adjacent distribution of the same type of residential areas in forms of concentric circle shaped, fan shaped and multi-centered real-life settlement, seven types of PSs in Dalian present a scattered status, the only exception being the cultural PSs with words related to campus and science in their residential quarters’ names. The two main reasons are as follows: (1) real-life settlements are the foundation of PSs. The uneven spatial distribution of certain elements in real-settlements influences the scattering of the corresponding types in PSs. For example, the differential spatial distribution of the hilly landform in Dalian causes the scattering of mountain-related developers’ brand culture; (2) The pursuit of innovation differentiation. For example, the coastal line of Dalian is quite long, but the spatial distribution of coastal PSs are not evenly distributed along the coastal line. Instead, they present an uneven situation of tripartite confrontation. This mainly results from spreaders’ pursuit of innovation effects and seeking differential features that attract the audience’s attention.

*Regional concentration*. PSs in Dalian are not completely scattered, they also present a phenomenon called regional concentration. The spatial concentration of certain elements in real-life settlements influence the concentration of the corresponding PSs. For example, the high-rise buildings in the real-life settlements mainly include the Qingniwaqiao Core Commercial Area in Zhongshan District and Xi’an Road Core Commercial Area in Shahekou District. The PSs of urban high-rise buildings retain their patterns, but they are still concentrated with these two commercial centers as the axis.

*Amplification and overflow effect of human settlement elements*. For example, the public infrastructures on Dalian Hongqi Subdistrict are greatly improved by virtue of the unique living environment of Xishan Reservoir and the spring breeze of “Western Expansion and Northern Development” in Dalian. This allows the community to become more mature. Thus Dalian Hongqi Subdistrict is a place strategically important for real estate developers such as Yida, Vanke, *etc.*, and has become the typical representative of mountain-related developers’ brands cultural PSs. To the north of Hongqi Subdistrict, Xinzhaizi Subdistrict also features a hilly landform, but its landscape reputation and popularity is not as great as that of the Xishan Reservoir area. However, due to its adjacent position, Xinzhaizi Subdistrict is also characterized as a mountain-related PS. This is because spreaders use the spreading medium to amplify its mountain household landscape features, in order to achieve the special purpose of publicity, inspiration and promotion [30].

## 4. Discussions and Conclusions

In this paper, we have proposed the concept of pseudo-settlement. A PS is a type of media information, which the media restructure, prompt and spread to people after selecting and processing housing information. In this way, audience can see the abundant and different urban human settlements all over the world through these media. A PS has the characteristics of universality, complexity, fragmentation, incompleteness, *etc.*

Using the names of residential quarters which are the most common, the most direct, the most likely to spread in various media, highly concentrated pseudo real-life settlements and easy to collect data as the study objectives, we established the PS index system of Dalian, consisting of the general names of residential quarters (pseudo-buildings) and special names (environment, region, culture and developer). The spatial features of each element of the obtained PSs of Dalian are as follows: the private and detached pseudo-residential-houses are mainly distributed in the areas with landscape advantages; the high-rise pseudo-buildings are highly concentrated in the downtown area; the large residential pseudo-building-clusters are mainly located in the new city area, followed by the old city area; courtyard pseudo-building-clusters are distributed the most widely distributed; the water related pseudo-settlements are mainly concentrated around Xinghai Bay and Eastern harbor; the mountain related pseudo-settlements are mainly located in the Xishan and Nanshan area; the flower related pseudo-settlements are mostly distributed in the old city area; the street related pseudo-settlements are mainly concentrated around the traditional old street areas and areas with symbolic significance, such as Zhonghua Road and Jinxiu Road; the western science and education cultural pseudo-settlements are mainly located in High-tech Zone, with the a tendency of spreading; the urban cultural pseudo-settlements area concentrated around the CBD and sub-CBD areas; the developers’ brands cultural pseudo-settlements are mainly located in the downtown area, expanding towards the north, west and southwest; the urban family related cultural pseudo-settlements are mainly located in the Shahekou District; and fortune related cultural pseudo-settlements are mainly located in Xinghai Bay and Zhongshan Square.

There are a total of eight types of PSs in Dalian. The spatial pattern features are regionally concentrated, widely scattered and there are places without pseudo-settlements. These features are mainly determined by the nature and the purposes of PSs. The nature and purpose of PSs are to affect change or complete and consolidate an audience’s imagined settlement. The places without PSs are evaluated by the spreaders as the areas having a minor impact on the audience and a lack of power to spread. Regional concentrated features mainly seek to properly amplify the local area's advantages to persuade the audience. The widely scattering feature seeks to draw public’s attention, filter the disadvantages, extract and amplify the optimal characteristics, and pursue innovation and differential results.

Urban PSs not only limit audience’s awareness, but also use information the audience is already aware of to affect real-life PSs. Boorstin [14] notes that the pseudo-event is often intended to be a self-fulfilling prophecy. PSs are the same, when PSs are regionally concentrated they will become more and more important. They deepen and solidify audience’s awareness and greatly affect the audience’s decisions toward activities like work, travel, planning, investing money, migrating and real estate development.

The universality and complexity of urban PSs determine the diversity and complicity of the study objects. Comprehensive study is required for the study of the urban PS and spatial patterns, namely various forms of spreading contents of various media shall be analyzed on a whole. Meanwhile, the detailed interpretation of single-form content spreading is also required. Only the clear and vigorous partial study can complete the comprehensive study accurately. This paper only selects the names of residential quarters spread through the media as the study objects. This method has certain limitations, but it is an essential part in the overall study of urban PSs. Future studies will focus on how to integrate the partial studies. Meanwhile, it is also necessary to consider different influences pseudo-settlements have on audiences of different age groups, genders and economic conditions. This paper evaluates the PSs’ degrees of deviation from real-life settlements and imagined settlements.

The diversity of urban PSs reflect the variety of human settlement cultures, and also indicates how people value philosophy, aesthetic pursuits and settlement preferences. This can helps guide urban management to improve real-life settlements. Urban PSs can also help administrators understand residents’ basic requirements and psychological needs concerning the human settlement environment. In addition, in consideration of the complicated system and enormous number of existing residential quarters’ names and its growth rate, property developers should increasing pay attention to the brands’ value awareness and promotion. Urban administrators should restrict and regulate the standardized names of the residential quarters. In the future, urban PSs study should focus on the relationship and differentiation between the urban PSs and real-life settlements, and analyse their similarity and deviations. The time dimension of urban PSs could also be taken into consideration. Urban PSs have been changing during the last 60 years since the founding of the People’s Republic of China. The variation of urban PSs leads to the changed perceptions about human settlements. Furthermore, the psychological mechanism and propagation process are other future research directions.

## References

[1] Chai Y. (2012). The Thoughts and Methods of Urban Geography.

[2] Lippmann W. (1946). Public Opinion.

[3] McCombs M.E., Shaw D.L. (1972). The agenda-setting function of mass media. Public Opin. Q..

[4] Wu L.Y. (2001). Introduction to Sciences of Human Settlements.

[5] Lynch K. (1997). The Image of the City.

[6] Tuan Y.F. (1975). Images and mental maps. Ann. Assoc. Am. Geogr..

[7] Bai K. (2009). A review of researches on tourism destination image positioning: A psychological perspective. Tourism Sci..

[8] Smith G.C. (1992). The cognition of shopping centers by the central area and suburban elderly: An analysis of consumer information fields and evaluative criteria. Urban Geogr..

[9] Li X., Xu X.Q. (1993). Analysis of urban image space in Guangzhou. Hum. Geogr..

[10] Gu C.L., Song G.C. (2001). Urban image space and main factors in Beijing. Acta. Geogr. Sin..

[11] Lippmann W. The World Outside and the Pictures in Our Heads. https://en.wikipedia.org/wiki/Public_Opinion_%28book%29.

[12] Cameron N. (1943). The Paranoid Pseudo-Community. Am. J. Sociol..

[13] Cameron N. (1959). The paranoid pseudo-community revisited. Am. J. Sociol..

[14] Daniel J.B. (2012). The Image: A Guide to Pseudo-Events in American.

[15] Beniger J.R. (1987). Personalization of mass media and the growth of pseudo-community. Commun. Res..

[16] Clarke J. (2003). How journalists judge the “reality” of an international “pseudo-event”. Journalism.

[17] Paterson C., Andresen K., Hoxha A. (2012). The manufacture of an international news event: The day Kosovo was born. Journalism.

[18] Hillman S. (2015). Empty-handed beauty: Juliette Récamier as pseudo-event. Celebrity Stud..

[19] Nedelea A., Năstase C., Κουρούπη-Κελγιαννάκη Γ. (2011). Tourism Potential and Marketing Strategies in Romanian Hospitality Industry. J. Tourism Res..

[20] Zhao Jianguo Pseudo-Environment and Human Being’s Cognition and Practices. http://d.wanfangdata.com.cn/Periodical/xwj200804037.

[21] Zhou Z. (2003). Media Architecture: The Thoughts about Architecture Design Giving by Media Broadcast.

[22] Jim B., Jack L., Andrew C.B., Kimberly R.S., Crystal L.H., Jeremy N. (2002). Immersive virtual environment technology as a methodological tool for social psychology. Psychol. Inq..

[23] Howard T., Gaborit N. (2007). Using virtual environment technology to improve public participation in urban planning process. Urban. Plan..

[24] Tong H. (2002). On the naming of the residential buildings and dwelling culture. J. Shanghai Univ..

[25] Li J., Shen T., Li X. (2013). The formation of Pseudo-environment and relationship between environment and Pseudo-environment. J. Liaoning Normal Univ..

[26] Wang Y., Zhao W. (2012). Information theory for human settlements research and its info-spectrum images system. Acta. Geogr. Sin..

[27] Liu P., Liu C., Deng Y. (2010). Landscape division of traditional settlement and effect elements of landscape gene in China. Acta Geogr. Sin..

[28] Li X., Li J. (2006). Analysis of urban space in Dalian. Acta Geogr. Sin..

[29] Chen L., Zhang W., Li Y. (2008). Urban residential suitability evaluation of Dalian’s residents. Acta. Geogr. Sin..

[30] Li X.M., Li H.H., Li J.H., Zhang Y.J. (2015). Geographical Research of Human Settlements from Positivism to Humanism.

